# Cannabinoid Receptor Type 1 Regulates Drug Reward Behavior via Glutamate Decarboxylase 67 Transcription

**DOI:** 10.3390/ijms221910486

**Published:** 2021-09-28

**Authors:** Sun Mi Gu, Sowoon Seo, Daejin Park, Sanghyeon Kim, Santosh Lamichhane, Kyoung-Moon Han, Young-Hoon Kim, Sangmin Lee, Jin Tae Hong, Hye Jin Cha, Jaesuk Yun

**Affiliations:** 1 College of Pharmacy, Chungbuk National University, 194-31, Osongsaengmyeong 1-ro, Osong-eup, Heungdeok-gu, Cheongju-si 28160, Chungcheongbuk-do, Korea; g09010327@nate.com (S.M.G.); olwg12@naver.com (S.S.); djpark08@naver.com (D.P.); jinthong@chungbuk.ac.kr (J.T.H.); 2 Stanley Brain Research Laboratory, Stanley Medical Research Institute, 9800 Medical Center Drive, Rockville, MD 20850, USA; obob1@naver.com; 3 College of Pharmacy, Wonkwang University, 460, Iksan-daero, Iksan-si 54538, Jeollabuk-do, Korea; lakki38786@gmail.com; 4 Center for Advanced Analysis, National Institute of Food and Drug Safety Evaluation (NIFDS), Ministry of Food and Drug Safety (MFDS), 187, Osongsaengmyong 2-ro, Osong-eup, Heungdeok-gu, Cheongju-si 28159, Chungcheongbuk-do, Korea; lovelyhkm@korea.kr; 5 Pharmacological Research Division, National Institute of Food and Drug Safety Evaluation (NIFDS), Ministry of Food and Drug Safety (MFDS), 187, Osongsaengmyong 2-ro, Osong-eup, Heungdeok-gu, Cheongju-si 28159, Chungcheongbuk-do, Korea; k1631@korea.kr; 6 Department of Pharmacy, College of Pharmacy, Kyung Hee University, 26 Kyungheedae-ro, Dongdaemun-gu, Seoul 02447, Korea; leesm@khu.ac.kr; 7 Deputy Director General for Narcotics Safety Planning, Pharmaceutical Safety Bureau, Ministry of Food and Drug Safety (MFDS), 187, Osongsaengmyong 2-ro, Osong-eup, Heungdeok-gu, Cheongju-si 28159, Chungcheongbuk-do, Korea

**Keywords:** cannabinoid receptor type 1, impulsivity, conditioned place preference, glutamate decarboxylase 67, methamphetamine

## Abstract

Interaction of cannabinoid receptor type 1 (CB1) and GABAergic neuronal activity is involved in drug abuse-related behavior. However, its role in drug-dependent Pavlovian conditioning is not well understood. In this study, we aimed to evaluate the effects of a CB1 agonist, JWH-210, on the development of conditioned place preference (CPP)-induced by methamphetamine (METH). Pretreatment with a synthetic cannabinoid, JWH-210 (CB1 agonist), increased METH-induced CPP score and METH-induced dopamine release in acute striatal slices. Interestingly, CB1 was expressed in glutamate decarboxylase 67 (GAD67) positive cells, and overexpression of CB1 increased GAD67 expression, while CB1 knockdown reduced GAD67 expression in vivo and in vitro. GAD67 is known as an enzyme involved in the synthesis of GABA. CB1 knockdown in the mice striatum increased METH-induced CPP. When GAD67 decreased in the mice striatum, mRNA level of *CB1* did not change, suggesting that CB1 can regulate GAD67 expression. GAD67 knockdown in the mouse striatum augmented apomorphine (dopamine receptor D2 agonist)–induced climbing behavior and METH-induced CPP score. Moreover, in the human brain, mRNA level of *GAD67* was found to be decreased in drug users. Therefore, we suggest that CB1 potentiates METH-induced CPP through inhibitory GABAergic regulation of dopaminergic neuronal activity.

## 1. Introduction

Cannabinoids act on the endocannabinoid system primarily through binding cannabinoid receptor type 1 (CB1), to modulate memory, emotions, and pain [1,2,3,4]. In the hotplate test, Δ^9^-Tetrahydrocannabinol (Δ^9^-THC), a representative cannabinoid accounting for the major psychoactive component in marijuana, induced analgesia in CB1^+/+^ mice, but not in CB1^−/−^ mice [5]. Administration of Δ^9^-THC also induced a significant decrease in spatial learning and memory in the radial arm maze; however, microinjection of rimonabant, a CB1 antagonist, dorsal to the hippocampus reduced memory impairment [5]. In a clinical study, exposure to Δ^9^-THC induced anxiety, which was correlated with CB1 expression [6]. Moreover, Δ^9^-THC from natural cannabinoids and synthetic cannabinoids have varying effects, which are typically similar to those of marijuana [7,8,9].

Centrally, marijuana affects the dopamine system, which is involved in motivation, reward, and recognition [10,11,12]. Synthetic cannabinoids are potent CB1 agonists and exert Δ^9^-THC–like effects [13]. Although dopaminergic neurons do not express CB1, activation of this receptor is involved in regulation of the dopamine system [14]. Several studies have observed that both synthetic and naturally-occurring cannabinoids induce dopaminergic neuron activity and dopamine release [14,15,16,17]. Furthermore, Δ^9^-THC treatment dose-dependently increases the activation of dopaminergic neurons in the ventral tegmental area (VTA). However, pretreatment with SR141716A, a CB1 antagonist, partially, or completely, inhibits Δ^9^-THC-induced dopaminergic neuron activation in the VTA [16]. Additionally, cocaine-induced dopamine release is attenuated in CB1^−/−^ mouse striatal slices compared with CB1^+/+^ mouse striatal slices [18]. Taken together, these studies indicate that CB1 may regulate dopaminergic neuron function. Moreover, prior treatment with Δ^9^-THC enhances subsequent nicotine self-administration in rats [19]. These “gateway drug effects” suggest that lasting changes to CB1 may play a key role in the rewarding effects of nicotine and other abused drugs [19].

CB1 is found on the terminals of central GABAergic and glutamatergic neurons in various brain regions where they mediate inhibition of neurotransmitter release [20,21,22,23]. Disrupted CB1 signaling is associated with psychological and emotional disorders including anxiety, depression, and schizophrenia [24,25]. Especially, decreased CB1 expression on GABAergic neurons results in increased impulsivity [26].

GABA is synthesized in GABAergic neurons from glutamate by the enzyme glutamic acid decarboxylase 1 or 2 (GAD1/GAD67 or GAD2/GAD65) [27]. While glutamate participates widely in cellular metabolism, including protein synthesis, GABA functions as a neurotransmitter. Hence, it is synthesized only in GABAergic neurons [28]. GABA can also inhibit the activity of dopaminergic neurons in the substantia nigra via the striatonigral and pallidonigral pathways [29]. Meanwhile, Δ9-THC decreases the extracellular levels of GABA in the prefrontal cortex of rats, while increasing glutamate and dopamine levels in this region [30].

There are hundreds of cannabinoid receptors agonists with varying affinities for CB1 that can be used as potential targets for addictive drugs [31]. In this study, we investigated whether the synthetic cannabinoid JWH-210, which has a high affinity for CB1, increases the dependence liability of other abused drugs, such as methamphetamine (METH) [32]. In addition, we also investigated how CB1 agonist affects neurotransmitters, such as dopamine, which are involved in impulsive behaviors and psychological dependence that may result from substance abuse.

## 2. Results

### 2.1. JWH-210 Binds to CB1 In Vivo

**Figure 1:**
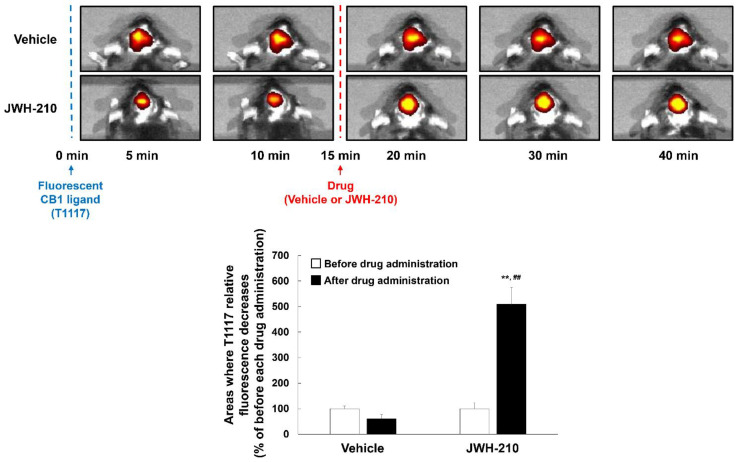
Displacement of fluorescent CB1 ligand binding in vivo by JWH-210. CB1 ligand (T1117; 10 μL, 5 mM in DMSO, i.c.v) was administered 15 min before JWH-210 (0.1 mg/kg, i.p.). Arrows indicate fluorescent CB1 ligand and drug (vehicle or JWH-210) injection time (0 and 15 min, respectively). Data are expressed as the mean ± S.E. (*n* = 2–3) and were analyzed using two-way ANOVA followed by Bonferroni *post-hoc t*-test (** *p* < 0.01 vs. each before drug administration group; ^##^ *p* < 0.01 vs. Vehicle/After drug administration group). I.C.V.: intracerebroventricular injection. I.P.: intraperitoneal injection.

Employing an in vitro receptor-binding assay, we previously reported that JWH-210 binds to CB1 [33]. However, to the best of our knowledge, there are no studies showing the interaction of JWH-210 and CB1 in vivo [34]. To determine if JWH-210 binds CB1 in vivo, we performed competitive binding assays using T1117, a fluorescent AM-251 analog. T1117 fluorescence is increased upon binding with CB1 and quenched upon dissociation. JWH-210 competes with T1117 for CB1 binding, and decreasing fluorescence signals from the unbound T1117 indicate specific CB1 binding of JWH-210 in competitive binding assays [35]. In this study, we found that intra-cerebrovascular administration of T1117 induced fluorescence signals that lasted at least 40 min. JWH-210 (0.1 mg/kg i.p.) injection increased areas where T1117 relative fluorescence decreases, while vehicle did not have any difference [Figure 1; CB1 ligand condition: F (1,6) = 24.824, *p* = 0.002; drug condition: F (1,6) = 16.845, *p* = 0.006; interaction: F (1,6) = 24.824, *p* = 0.002]. These results indicate that JWH-210 binds to CB1 in vivo, and its binding strength is strong.

### 2.2. JWH-210 Exhibits CB1 Agonist Activity

**Figure 2:**
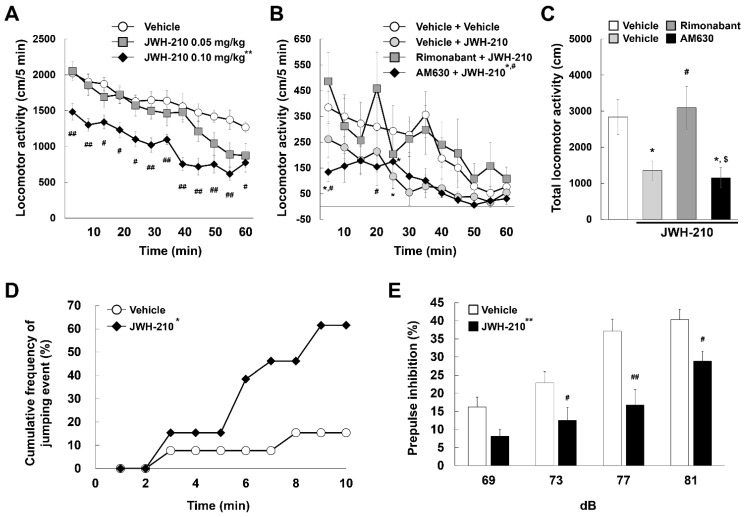
Effects of JWH-210 on behavioral tests. (**A**) Mice were injected with either vehicle or JWH-210 (0.05 or 0.1 mg/kg, i.p.). Locomotor activity was measured at 5 min intervals for 60 min. Data are expressed as the mean distance traveled ± S.E. (*n* = 10) and were analyzed using two-way RM ANOVA followed by Bonferroni *post-hoc t*-test [** *p* < 0.01 vs. vehicle group; ^#^ *p* < 0.05 and ^##^ *p* < 0.01 vs. vehicle group (at the same time point)]. (**B**,**C**) Mice were injected with either vehicle or rimonabant (CB1 antagonist, 1 mg/kg) or AM630 (CB2 antagonist, 3 mg/kg) 30 min prior to JWH-210 (0.1 mg/kg, i.p.) injection. (**B**) Locomotor activity was measured at 5 min intervals for 60 min. Data are expressed as the mean distance traveled ± S.E. (*n* = 6–7) and were analyzed using two-way RM ANOVA followed by Bonferroni *post-hoc t*-test [* *p* < 0.05 vs. vehicle + vehicle group (at the same time point); ^#^ *p* < 0.05 vs. rimonabant + JWH-210 group (at the same time point)]. (**C**) Total locomotor activity was measured for 60 min. Data are expressed as the mean total distance traveled ± S.E. (*n* = 6–7) and were analyzed using one-way ANOVA followed by Holm–Sidak *post-hoc t*-test (* *p* < 0.05 vs. vehicle + vehicle group; ^#^ *p* < 0.05 vs. vehicle + JWH-210 group; ^$^ *p* < 0.05 vs. rimonabant + JWH-210 group). (**D**,**E**) Mice were injected with either a vehicle or JWH-210 (0.1 mg/kg, i.p.) once every day for 5 days, and then behavior tests were performed. (**D**) The cumulative frequency of jumping events (%) was measured by CAR test. Data are expressed as the differences between vehicle group and JWH-210 group curves, and were analyzed using Fisher’s exact test (* *p* < 0.05). (**E**) The sensorimotor gating was measured by PPI test. Data are expressed as the mean ± S.E. (*n* = 10) and were analyzed using two-way RM ANOVA followed by Bonferroni *post-hoc t*-test [** *p* < 0.01; ^#^ *p* < 0.05 and ^##^ *p* < 0.01 vs. vehicle group (on the same dB)].

A previous study showed that acute administration of CB1 agonists suppresses locomotor activity in animals [36]. Therefore, we examined whether JWH-210 affects locomotor activity. A single administration of 0.1 mg/kg JWH-210 significantly reduced the locomotor activity compared to vehicle [Figure 2A; drug condition: F (2,27) = 10.351, *p* < 0.01; time: F (11,297) = 41.08, *p* < 0.01; interaction: F (22,297) = 1.830, *p* = 0.014]. Furthermore, we investigated which CB receptor is associated with JWH-210-induced hypolocomotion. To this end, mice were treated with rimonabant (CB1 antagonist, 1 mg/kg, i.p.) or AM630 (CB2 antagonist, 3 mg/kg, i.p.) prior to JWH-210 (0.1 mg/kg, i.p.) injection and locomotor activity test was subsequently measured. Results show that JWH-210-induced hypolocomotion was recovered by rimonabant, while AM630 did not interfere with the effect elicited by JWH-210 [Figure 2B; drug condition: F (3,23) = 5.279, *p* = 0.006; time: F (11,253) = 9.882, *p* < 0.01; interaction: F (33,253) = 0.84, *p* = 0.72] [Figure 2C; F (3,23) = 5.708, *p* = 0.005]. These results suggest that JWH-210 functions as a CB1 agonist in vivo.

### 2.3. Five-Day Administration of JWH-210 Affects Impulsivity and Sensorimotor Gating

We performed CAR assays to investigate changes in JWH-210-induced impulsivity. Administration of JWH-210 for five days caused mice to jump off a platform significantly more than control mice within the ten-minute assay period. Eight of thirteen JWH-210-pretreated mice jumped, while two of thirteen vehicle-pretreated mice jumped, therefore, administration of JWH-210 significantly increased impairment of CAR (Figure 2D; Fisher’s exact test, *p* = 0.041). Increased jumping induced by JWH-210 was inhibited by pretreatment with rimonabant (Figure S3). These results suggest that JWH-210 increases impulsivity. Cannabinoid abuse is related to schizophrenia and abnormal sensorimotor gating responses in PPI assays. Administration of JWH-210 for five days significantly decreased PPI compared to vehicle administration [Figure 2E; drug condition: F (1,18) = 17.144, *p* < 0.01; dB: F (3,54) = 28.337, *p* < 0.01; interaction: F (3,54) = 2.097, *p* = 0.111]. These results indicate that JWH-210 decreases sensorimotor gating.

### 2.4. Pretreatment of JWH-210 Increases METH-Induced CPP

**Figure 3:**
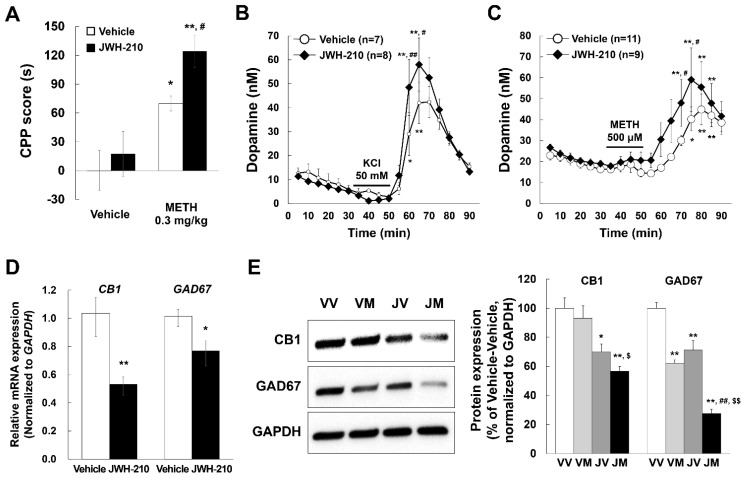
Effects of JWH-210 on METH-induced CPP, KCl-induced dopamine release, or METH-induced dopamine efflux and CB1 and GAD67 expression in mice striatum. Before starting the CPP test, acute brain slices, and dissecting the brain, mice were injected with either a vehicle or JWH-210 (0.1 mg/kg, i.p.) once every day for 5 days. (**A**) 0.3 mg/kg METH-induced CPP scores (s) was measured by CPP test. Data are expressed as the mean ± S.E. (*n* = 13) and were analyzed using two-way ANOVA followed by Bonferroni *post-hoc t*-test (* *p* < 0.05 and ** *p* < 0.01 vs. each vehicle group; ^#^ *p* < 0.05 vs. vehicle/METH group). (**B**) 50 mM KCl-induced dopamine release or (**C**) 500 μM METH-induced dopamine efflux in brain striatal slices were measured by using HPLC. Data are expressed as the mean ± S.E. (*n* = number of mice) and were analyzed using two-way RM ANOVA followed by Bonferroni *post-hoc t*-test [* *p* < 0.05, ** *p* < 0.01, and ** *p* < 0.01 vs. each basal level (at 30 min); ^#^ *p* < 0.05 and ^##^ *p* < 0.01 vs. vehicle group on the same time]. (**D**) Mice striatum were collected after 1 day following the final injection. mRNA levels of *CB1* and *GAD67* were detected by qPCR and normalized relative to the amplification of GAPDH in the mice striatum. Data are expressed as the mean ± S.E. (*n* = 5–9, Student’s *t*-test; * *p* < 0.05 and ** *p* < 0.01 vs. vehicle group). (**E**) Mice striatum were collected after CPP test. The protein expression of CB1 and GAD67 was detected by Western blotting using specific antibodies and normalized to the relative amplification of GAPDH. Data are expressed as the mean total distance traveled ± S.E. (*n* = 5) and were analyzed using one-way ANOVA followed by Holm–Sidak *post-hoc t*-test (* *p* < 0.05 and ** *p* < 0.01 vs. VV group; ^##^ *p* < 0.01 vs. VM group; ^$^ *p* < 0.05 vs. JV group, ^$$^ *p* < 0.01 vs. JV group). METH: methamphetamine. VV: vehicle + vehicle group. VM: vehicle + methamphetamine group. JV: JWH-210 + vehicle group. JM: JWH-210 + methamphetamine group.

To assess the effects of JWH-210 on the development of Pavlovian conditioning induced by METH, we performed CPP assays. METH (0.3 mg/kg) significantly induced CPP in mice pretreated with vehicle or JWH-210 (0.1 mg/kg, for 5 days), moreover, mice pretreated with JWH-210 showed even higher CPP than mice pretreated with vehicle [Figure 3A; pretreatment drug condition: F (1,36) = 3.808, *p* = 0.059; CPP drug condition: F (1,36) = 23.206, *p* < 0.01; interaction: F (1,36) = 0.988, *p* = 0.327]. However, the 1 mg/kg METH-induced CPP did not differ significantly from METH-induced CPP following JWH-210 pretreatment, possibly because 1 mg/kg METH already induced the maximum CPP (Figure S4A). Moreover, pre-administration of JWH-210 also increased CPP score induced by para-chloroamphetamine (PCA), which is a substituted amphetamine (Figure S4B). These results indicate that JWH-210 increases METH-induced Pavlovian conditioning.

### 2.5. JWH-210 Treatment Increases KCl-Induced Dopamine Release and METH-Induced Dopamine Efflux

METH induces dopamine release and thereby increases the extracellular dopamine concentration in the striatum. METH-induced enhancement of dopamine release is associated with behavioral alterations such as CPP [37]. Furthermore, highly impulsive rats are more sensitive to the rewarding effects of stimulants such as amphetamine [38]. Additionally, striatal dopamine release is associated with impulsivity and sensorimotor gating [39]. Therefore, we investigated whether JWH-210 is involved in regulating dopamine levels in the striatum. While basal dopamine levels were not different between JWH-210- and vehicle-treated mice, KCl-induced dopamine release was higher in brain slices of mice treated with JWH-210 compared to vehicle-treated mice [Figure 3B; pretreatment drug condition: F (1,13) = 0.462, *p* = 0.509; Time: F (17,221) = 31.246, *p* < 0.01; interaction: F (17,221) = 1.425, *p* = 0.126.]. JWH-210 administration also augmented METH-induced dopamine release [Figure 3C; pretreatment drug condition: F (1,18) = 3.170, *p* = 0.092; Time: F (17,306) = 12.939, *p* < 0.01; interaction: F (17,306) = 0.908, *p* = 0.565]. Taken together, these findings suggest that JWH-210 increases KCl-induced dopamine release and METH-induced dopamine efflux.

### 2.6. Administration of JWH-210 Reduced CB1 Expression in Mice Striatum

To identify the mechanism underlying increased dopamine release in JWH-210 pretreated mice, we measured the levels of dopamine-related proteins. JWH-210 treatment did not have any effects on the levels of dopamine-related enzymes, including *Th* (tyrosine hydroxylase), *Slc6a3* (dopamine transporter), *Slc18a2* (vesicular monoamine transporter 2), *Drd1a* (dopamine receptor D1a), *Drd2* (dopamine receptor D2), *Maoa* (monoamine oxidase A), and *Maob* (monoamine oxidase B) in the striatum (Table S1). Given that we found that CB1 are expressed on GABAergic neurons but not dopaminergic neurons (Figure S5), we investigated whether JWH-210 treatment affects CB1 and GAD67 expression. To assess the changes in *CB1* mRNA level in mice treated with JWH-210 for five days, we conducted qPCR using mice striatum samples. One day after the final JWH-210 injection, mRNA levels of *CB1* and *GAD67* decreased (Figure 3D). Further, protein expression of CB1 and GAD67 was also reduced in the striatum of JWH-210-treated mice as compared to that of the vehicle-treated mice, especially, these expressions were further decreased in JWH-210/METH-treated mice [Figure 3E; F (3,8) = 48.064, *p* < 0.001]. These results suggest that five-day treatment with JWH-210 decreases CB1 expression in GABAergic neurons.

### 2.7. CB1 Regulates GAD67 Expression in Cultured Primary Neurons

**Figure 4:**
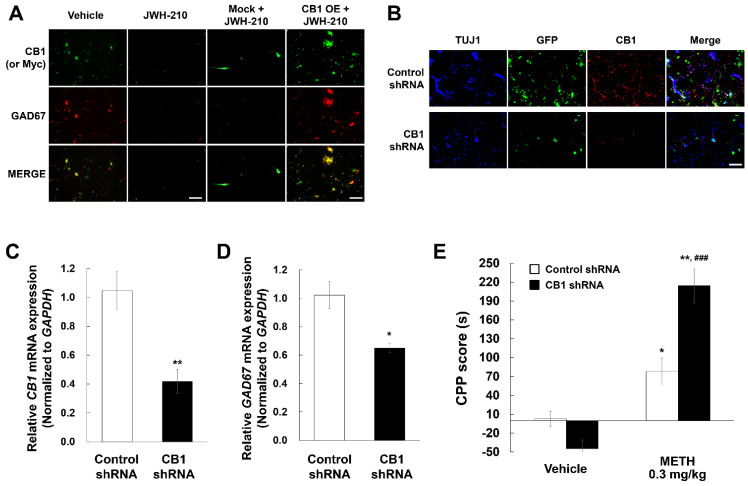
Effects of CB1 knockdown on GAD67 expression in primary cultured neurons and METH-induced CPP. (**A**) Vehicle group was treated with vehicle (5% DMSO, 5% tween-20, 90% PBS) as the same amount of JWH-210 for 24 h. JWH-210 group was treated with 0.1 μM JWH-210 for 24 h. Mock + JWH-210 and CB1 overexpression (OE) + JWH-210 groups were treated with mock or CB1 ORF lentiviral particles (5 MOI) for 24 h before treatment with vehicle or 0.1 μM JWH-210 for 24 h. Immunostaining of CB1, Myc, and GAD67 was performed with specific antibodies (Scale bar: 200 μm). (**B**) Cultured neurons were treated with control or CB1 shRNA lentiviral particles. mRNA levels of (**C**) *CB1* and (**D**) *GAD67* were confirmed by using qPCR with each specific primer and normalized to the relative amplification of GAPDH. Data are expressed as the mean ± S.E. (*n* = 6; Student’s *t*-test; * *p* < 0.05 and ** *p* < 0.01 vs. vehicle group). (**E**) After CB1 knockdown, 0.3 mg/kg METH-induced CPP scores (s) was measured by CPP test. Data are expressed as the mean ± S.E. (*n* = 8) and were analyzed using two-way ANOVA followed by Bonferroni *post-hoc t*-test (* *p* < 0.05 and ** *p* < 0.01 vs. each saline group; ^#^^##^ *p* < 0.001 vs. Control shRNA/METH group). OE: overexpression. TUJ1: beta-tubulin III. METH: methamphetamine.

To investigate the role of CB1 in regulating GAD67 levels, we overexpressed or knocked down CB1 in cultured mouse primary neurons. The cultured neurons were transduced using CB1 ORF lentiviral particles and treated with 0.1 µM JWH-210 for 24 h. The expression of GAD67 and CB1 were lower in JWH-210-treated primary neurons compared to vehicle-treated neurons (Figure 4A). Further, GAD67 expression was rescued by CB1 overexpression (Figure 4B). For the knockdown experiment, we treated the cultured neurons with CB1 shRNA and measured mRNA level of *GAD67*. CB1 shRNA treatment reduced CB1 levels (Figure 4C) and suppressed mRNA level of *GAD67* (Figure 4D). These results suggest that CB1 plays a role in regulating GAD67 expression.

### 2.8. CB1 Knockdown Increases METH-Induced CPP

To assess the role of CB1 and GAD67 in the development of METH-induced Pavlovian conditioning, we performed CPP assays. METH-induced CPP was higher in the CB1 knockdown mice compared to control-injected mice [Figure 4E; CB1 condition: F (1,28) = 5.230, *p* = 0.030; CPP drug condition: F (1,28) = 75.056, *p* < 0.01; interaction: F (1,28) = 22.514, *p* < 0.01]. These results indicate that CB1 regulates METH-induced Pavlovian conditioning.

### 2.9. Apomorphine-Induced Climbing Behavior and METH-Induced CPP Increase with GAD67 Knockdown

**Figure 5:**
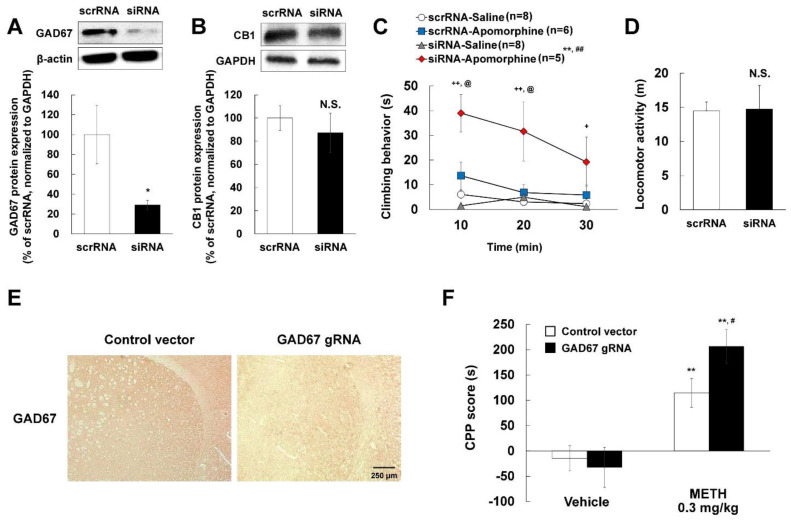
Effects of GAD67 knockdown on CB1 expression in mice striatum, apomorphine-induced climbing behavior, locomotor activity, and METH-induced CPP. Mice were injected with either scrRNA or GAD67 siRNA by stereotaxic injection into the right striatum. Experiments were started 3 days after stereotaxic injection. The protein expression of (**A**) GAD67 and (**B**) CB1 was detected by Western blotting with each specific antibody and normalized to β-actin or GAPDH. Data are expressed as the mean ± S.E. (*n* = 4–5 for each group; Student’s *t*-test; * *p* < 0.05 and not significant (N.S.) vs. vehicle group). (**C**) Climbing behavior was measured after administration of saline or apomorphine (1 mg/kg, i.p.). Data are expressed as the mean ± S.E. (*n* = number of mice) and were analyzed using three-way RM ANOVA and two-way RM ANOVA followed by Bonferroni *post-hoc t*-test (** *p* < 0.01 vs. siRNA-Saline group, ^##^ *p* < 0.01 vs. scrRNA-Apomorphine group, ^++^ *p* < 0.01 and ^+^ *p* < 0.05 vs. siRNA-Saline group in each time zone, ^@^ *p* < 0.05 vs. siRNA-Apomorphine group in each time zone). (**D**) Locomotor activity was measured for 60 min without any drug challenge. Data are expressed as the mean ± S.E. (*n* = 5–6 for each group; Student’s *t*-test; not significant (N.S.) vs. vehicle group). (**E**) The mice were stereotaxically injected with control or GAD67 gRNA CRISPR/Cas9 vectors. The mice striatum sections (10-μm-thick) were reacted with GAD67 antibody. Original magnification is ×200. (**F**) After GAD67 knockdown, 0.3 mg/kg METH-induced CPP scores (s) was measured by CPP test. Data are expressed as the mean ± S.E. (*n* = 9–14 for each group) and were analyzed using two-way ANOVA followed by Bonferroni *post-hoc t*-test (* *p* < 0.05 and ** *p* < 0.001 vs. each saline group; ^#^ *p* < 0.05 vs. Control vector/METH group). METH: methamphetamine.

The dopamine receptors D1 and D2 play a critical role in the development of CPP-induced by abused drugs [40,41,42,43]. Therefore, to elucidate the role of GAD67 in regulating dopamine receptor activity, we performed apomorphine-induced climbing behavior tests in GAD67 knockdown mice. Apomorphine is a dopamine D1 and D2 receptor agonist and climbing behavior represents a unique response to striatal dopamine receptor stimulation. First, we confirmed that the levels of GAD67 were reduced in the striatum three days after GAD67 siRNA injection (Figure 5A). Importantly, the level of CB1 was unchanged (Figure 5B). GAD67 knockdown mice showed increased apomorphine-induced climbing behavior [Figure 5C; GAD67 condition: F (1,75) = 16.898, *p* < 0.01; climbing behavior drug condition: F (1,75) = 44.920, *p* < 0.01; Time: F (2,75) = 3.609, *p* = 0.032; GAD67 condition × climbing behavior drug condition interaction: F (1,75) = 21.652, *p* < 0.01; GAD67 condition × time condition interaction: F (2,75) = 0.772, *p* = 0.466; climbing behavior drug condition × time condition interaction: F (2,75) = 2.030, *p* = 0.138; GAD67 condition × climbing behavior drug condition × time interaction: F (2,75) = 0.839, *p* = 0.436]. Meanwhile, GAD67 knockdown mice had no changes in locomotor activity compared with control mice (Figure 5D). Therefore, these results suggest that GABAergic dysfunction plays a role in dopaminergic neuronal activity in the striatum. To evaluate a role of GAD67 in the development of Pavlovian conditioning induced by METH, we conducted additional CPP assays. Stereotaxic injection of GAD67 CRISPR/Cas9 gRNA vector decreased GAD67 expression in the striatum (Figure 5E). Furthermore, our vector was expressed in D1 and D2 positive striatal medium spiny neuron (Figure S6). These results suggest that direct or indirect pathways are disrupted by GAD67 knockdown. However, GAD67 knockdown alone did not impact CPP, rather METH-induced CPP was increased in GAD67 knockdown mice [Figure 5F; GAD67 condition: F (1,37) = 1.357, *p* = 0.251; CPP drug condition: F (1,37) = 33.397, *p* < 0.01; interaction: F (1,37) = 2.968, *p* = 0.093], indicating that GAD67 is involved in METH-induced Pavlovian conditioning.

### 2.10. GAD67 Levels in the Human Frontal Cortex Are Significantly Correlated with Drug Use Severity Ratings

**Figure 6:**
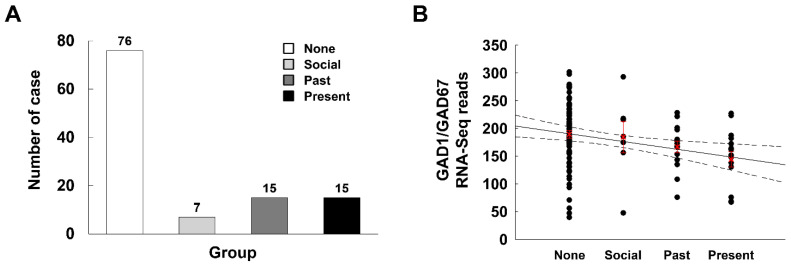
Correlation between GAD1/GAD67 gene expression in the human brain and the drug use severity ratings. (**A**) The number of cases in each drug use severity ratings. (**B**) The mean RNA-sequencing read counts of GAD1/GAD67 gene in each severity ratings. Data are expressed as raw data (●) and mean ± S.E. (X, red), and were analyzed using Pearson correlation test. The normal line is the plot of regression analysis and the dotted lines represent the 95% confidence intervals for the regression line.

To study the correlation between GAD67 and drug use, we performed RNA-seq analysis in samples from the frontal cortex of drug users. The samples were grouped as follows: “None” indicates no history of drug use, “Social” indicates a history of light drug use, “Past” denotes a history of moderate or heavy drug use in the past, and “Present” describes a history of recent moderate or heavy drug use (Figure 6A). GAD67 levels were lower in groups with a history of drug use compared to the group without prior drug use (Figure 6B). The major biological processes (gene ontology) significantly enriched in the genes that correlated with the severity ratings are listed in Table S2. These results suggest that GAD67 is associated with drug abuse.

## 3. Discussion

Our previous reports using CPP and self-administration tests suggest that JWH-210 has a rewarding effect [34]. Moreover, JWH-210 administration augmented METH-induced CPP in the present study, which may be associated with Pavlovian conditioning [44]. Cannabis treatment can predispose individuals to substance dependence and have long-lasting changes on the response to other drugs [19]. For example, cannabis exposure increases the sensitization and self-administration of heroin and morphine [45]. Our results suggest that JWH-210 enhances the rewarding effects of other abused drugs, such as METH. Abused drugs, including cannabis, can increase synaptic dopamine levels in the striatum, possibly mediating the motivating and rewarding effects of these compounds [46,47,48]. Interestingly, we reported that JWH-210 induces CPP at doses of 0.05 and 0.1 mg/kg, while higher doses, such as 0.5 and 1.0 mg/kg, induce conditioned place aversion [34]. Furthermore, we previously demonstrated that dopamine release in the nucleus accumbens is increased by 0.1 mg/kg JWH-210; however, is decreased at a dose of 1.0 mg/kg [49]. In the present study, pretreatment with 0.1 mg/kg JWH-210 augmented KCl-induced dopamine release and METH-induced dopamine efflux in mouse striatum slices.

Dopamine upregulation in the striatum is associated with impulsivity. Specifically, ventral striatal dopamine transporter availability is associated with lower trait motor impulsivity in healthy adults [50]. Impulsivity may then contribute to increased vulnerability to drug abuse [51]. In the present study, JWH-210 increased impulsivity in mice, evidenced by more frequent jumping events in the CAR assays. These results suggest that JWH-210 has a stimulatory effect on dopaminergic neuronal transmission at doses capable of inducing CPP and impulsivity.

CB1 mediates the behavioral and psychoactive effects of Δ^9^-THC in animals and humans [34,52,53]. Alterations in CB1 expression levels may mediate the long-term effects of cannabinoids [54]. We demonstrated that mRNA and protein levels of CB1 in the mouse striatum are decreased by JWH-210 administration. Furthermore, we showed that JWH-210-induced hypolocomotion is associated with the CB1 receptor. GAD67 was also reduced by JWH-210 injection, and exposure of METH further decreased these genes levels. The relationship between CB1 and GAD67 has been demonstrated in several previous studies. Δ^9^-THC reduces CB1, GAD67, and GABA levels in the prefrontal cortex of rats [54]. CB1 and GAD67 are co-expressed in mouse and rat striatal neurons [55]. In our study, CB1 and GAD67 were co-localized in primary cultured striatal neurons, and CB1 and parvalbumin were co-localized in primary cultured VTA neurons (Figure S5B). Parvalbumin is expressed in GABAergic interneurons and VTA GABA interneuron are implicated in reward consumption [56]. Therefore, reduced CB1 expression in GABAergic neurons may mediate the effect of JWH-210 on the dopaminergic system. We observed that GAD67 and CB1 expression were reduced by JWH-210, but GAD67 levels were rescued with CB1 overexpression in primary cultured neurons. Interestingly, when CB1 was knocked down in primary cultured neurons, mRNA level of GAD67 also decreased; however, the reciprocal relationship was not observed. Meanwhile, although the precise mechanisms underlying the regulation of CB1 on GAD67 levels remains unclear, it is well known that CB1 activation decreases GABAergic neurotransmission, therefore, CB1 reduction by synthetic cannabinoid administration may induce homeostatic regulation of GAD67 expression. Taken together, these results suggest that JWH-210-induced reduction of CB1 in GABAergic neurons decreased GAD67 and disrupted inhibitory regulation of dopaminergic neurons in striatum, consequently increasing dopamine release.

Reduced GABA or GAD67 levels are reportedly involved in mental disorders, such as schizophrenia, and substance use disorders [57,58,59]. In this study, GAD67 levels were observed to be lower in groups with a history of drug use compared to those with no prior use. In previous studies, inhibition of the indirect pathway from striatum to VTA and SN was reported to cause dopamine neuronal activation induced by abuse drugs [60,61]. In our study, we suggest that GAD67 knockdown disrupts the D2-mediated indirect pathway through the GABAergic interneuron, at least in part, in SN and, consequently, increases dopamine release. Low expression levels of GAD67 could disinhibit dopaminergic neuronal activity, consequently increasing dopamine release-induced by abused drugs. Therefore, reduced GAD67 levels may be associated with vulnerability to drug abuse. Our in vivo study also revealed that CB1 or GAD67 knockdown led to increased METH-induced CPP, suggesting that decreased CB1 levels may play a role in METH-induced CPP and in elevated dopamine release via regulation of GAD67 expression. In addition, knocking down GAD67 in the striatum increased apomorphine-induced climbing behavior. Since postsynaptic dopamine receptor activation in striatum evokes climbing behavior, decreased GAD67 levels may affect the formation of postsynaptic dopamine receptor sensitization as well as influence presynaptic dopamine release.

Taken together, we suggest that CB1 may play a role in the regulation of GAD67 expression through dopamine D2 receptor. Thus, JWH-210 may increase vulnerability to stimulants in mice through the regulation of dopamine release and dopamine receptor sensitization, which is likely related to reduced expression of CB1 and GAD67.

## 4. Materials and Methods

### 4.1. Animals

All experimental procedures were approved by the Animal Ethics Committee and the National Institute of Food and Drug Safety Evaluation (1601MFDS-10) and complied with the National Institutes of Health Guide for the Care and Use of Laboratory Animals (National Research Council, NRC, 1996). Male C57BL/6J mice (7 weeks old) were obtained from Charles River Laboratories Japan (Yokohama, Japan). Experiments began after a 1-week acclimatization period. Animal holding rooms were maintained at a temperature of 21–24 °C and 40–60% relative humidity with a 12-h light/dark cycle (lights on 08:00 to 20:00). The animals received a solid diet and tap water ad libitum.

### 4.2. Drugs

The synthetic cannabinoid JWH-210 (Figure S1) was purchased from Cayman Chemical (Ann Arbor, MI, USA). METH hydrochloride (HCl), rimonabant HCl, apomorphine HCl, AM630 were obtained from Sigma-Aldrich (St. Louis, MO, USA), and were dissolved in saline immediately prior to the experiments. JWH-210 was dissolved in vehicle (saline containing 5% Tween 80 and 5% DMSO), (R)-(+)-limonene (Sigma-Aldrich, St. Louis, MO, USA) was dissolved in saline containing 4% Tween 80, and apomorphine was dissolved in saline containing 0.1% ascorbic acid immediately prior to use. Other chemicals were obtained from Sigma-Aldrich (St. Louis, MO, USA) unless otherwise noted.

### 4.3. In Vivo Imaging

To investigate the distribution of JWH-210 on the central nervous system, we conducted imaging analysis using T1117, a fluorescent form of the CB1 ligand AM-251 (Bio-Techne Corporation, Minneapolis, MN, USA). Mice were anesthetized with pentobarbital (50 mg/kg, i.p.) and placed in a stereotaxic apparatus. The stereotaxic coordinates of the cerebral ventricle were 0.3 mm anterior to bregma, 1.0 mm lateral to the sagittal suture, and 2.5 mm ventral to the brain surface. T1117 was injected into the right cerebral ventricle (intracerebroventricular injection; 10 μL, 5 mM in DMSO). Fifteen minutes later, JWH-210 was injected intraperitoneally (i.p., 0.1 mg/kg body weight). The fluorescent signal was measured using an IVIS spectrum in vivo imaging system (PerkinElmer, Waltham, MA, USA). The signal was measured in the presence and absence of JWH-210. While imaging was in progress, the mice remained unconscious and mice’s heads were immobilized. The signal area was targeted to a specific color using Adobe Photoshop^®^ CC 2018 (Adobe, Park Avenue, CA, USA). The value of the signal area was measured using the ImageJ 1.53k (Wayne Rasband, National Institutes of Health, Bethesda, MD, USA) and was the percentage based on the value for 10 min prior to vehicle administration.

### 4.4. In Vivo CB1 and GAD67 Knockdown

Mice were injected with control or CB1 shRNA lentiviral particles (GFP tagged, Origene, Rockville, MD, USA), scramble or GAD67 siRNA (siRNA No. 1360538, 1360539, and 1360540; Bioneer, Daejeon, Korea), control or GAD67 CRISPR/Cas9 gRNA vector (eGFP tagged, Macrogen, Seoul, Korea) to induce CB1 or GAD67 knockdown. Mice were anesthetized with pentobarbital (50 mg/kg, i.p.) and placed in a stereotaxic apparatus. The stereotaxic coordinates of the striatum were 0.2 mm anterior to bregma, 2.0 mm lateral to the sagittal suture, and 4.5 mm ventral to the brain surface. Seven days before siRNA injection, a stainless-steel guide cannula (AD-8, Eicom, Tokyo, Japan) was implanted into the brain at the above coordinates. Mice were injected at a rate of 1 μL/min with a 10 μL Hamilton microsyringe as follows: control or CB1 shRNA delivery solution (5.4 × 104 TU shRNA in 2 μL), scramble or GAD67 siRNA delivery solution [300 pM siRNA with jetSI (Polyplus Transfection, New York, NY, USA) in 1 μL], or control or GAD67 gRNA delivery solution [0.4 μg vector with in vivo-jetPEI (Polyplus Transfection, New York, NY, USA) in 1.5 μL]. After injecting the solution, wait 1 min, then slowly raise the needle and remove it from the brain. The hole in the skull was closed using dental acrylate (Ortho-jet Lang Dental Manufacturing Company, Wheeling, IL, USA), and the incision site was closed using tissue adhesives (3M™ Vetbond Tissue Adhesive: 1469SB, 3M, St. Paul, MN, USA). Antibiotic ointment was applied to the incision site once every 2 days. After recovery for 7 days, the mice were subjected to behavioral tests.

### 4.5. Locomotor Activity

To investigate the effects of cannabinoids on locomotor activity, mice were administered vehicle or JWH-210 (0.05 or 0.1 mg/kg, i.p.). The drugs were administered without adaptation in the test cage. Locomotor activity was immediately measured at 5 min intervals for 60 min using an automatic tracking system (Panlab, Barcelona, Spain). To investigate the mechanism underlying JWH-210-induced hypolocomotion, mice were treated with vehicle or rimonabant (CB1 antagonist, 1 mg/kg, i.p.) or AM630 (CB2 antagonist, 3 mg/kg, i.p.). Mice were adapted in the test cage for 30 min after vehicle or antagonists administration. Locomotor activity was immediately measured at 5 min intervals for 60 min after administration of vehicle or JWH-210 (0.1 mg/kg, i.p.) to mice. To investigate the effects of GAD67 knockdown on mouse movement, mice were injected with the scrRNA or GAD67 siRNA stereotaxically as described above. After adaptation to the test cage for 60 min, locomotor activity was measured at 10 min intervals for 60 min using an automatic tracking system.

### 4.6. Cliff Avoidance Reaction Test

To evaluate impulsivity, we performed the cliff avoidance reaction (CAR) test. Mice were treated with either vehicle or rimonabant HCl (CB1 antagonist, 1 mg/kg, i.p.) 30 min before treatment with vehicle or JWH-210 (0.1 mg/kg, i.p.) once per day for 5 days. On day 6, cliff avoidance and jumping events were evaluated in the CAR test. Mice were placed on a round platform (an inverted glass container 13 cm wide and 20 cm tall), and behavior was measured for 10 min.

### 4.7. Prepulse Inhibition Test

Cannabinoids may impair sensorimotor gating functions. Therefore, we studied the effect of JWH-210 on prepulse inhibition (PPI). Mice were treated with either vehicle or JWH-210 (0.1 mg/kg, i.p.) once per day for 5 days. On day 6, PPI was evaluated using a startle/PPI box (Panlab, Barcelona, Spain) (Figure S2A). The mice were allowed to habituate for 10 min with 65 dB background noise. The PPI trials consisted of a prepulse (20 ms burst of 69-, 73-, 77-, or 81-dB white noise), followed by the startle stimulus (120 dB, 40 ms white noise) 100 ms after the prepulse. Each of the four prepulse trials (69, 73, 77, or 81 dB) were presented 10 times. There are 6 type of trials, which are 4 different prepulse-pulse trials, the startle pulse alone trials, the prepulse alone trials to be pseudorandomized and displayed 10 times each. The 60 different trials were performed pseudo-randomly, ensuring that each trial was presented 10 times and that no two consecutive trials were identical. The resulting movement of the animal in the startle chamber was measured for 100 ms after the startle stimulus onset (sampling frequency 1 kHz). Data were rectified, amplified, and recorded by a computer, which calculated the maximal response over the 100-ms period.

### 4.8. Conditioned Place Preference Test

To elucidate the effect of drug reward behavior, we performed CPP test with unbiased and counterbalanced trials. The experiment consisted of three methods in pretreatment stage, and CPP tests consisted of four phases (Figure S2B): (1) pre-conditioning phase, (2) pre-test phase, (3) conditioning phase, and (4) post-test phase. The time spent in each compartment was recorded and used to determine the preference for each compartment for 15 min. The CPP scores (s) were calculated from the changes between the post-test and pre-test phases.

### 4.9. Acute Brain Slices and Dopamine Measurement

To confirm the association between dopamine release and augmented CPP, we measured dopamine release in acute brain slices. Mice were treated with vehicle or JWH-210 (0.1 mg/kg, i.p.) once per day for 5 days (Figure S2A). On day 6, striatal slices (400 µm) were prepared using a tissue chopper. Slices were washed with chilled saline (pH 7.4) and perfused in a Brain/Tissue Slice Chamber System (BSC-ZT, Harvard Apparatus, Holliston, MA, USA) with Krebs-Henseleit buffer (K-H buffer; 1.2 mM KH2PO4, 2.5 mM NaHCO3, 1.2 mM MgSO4·7H2O, 11.7 mM D-glucose, 4.2 mM KCl, and 10 μM pargyline) bubbled with 95% O_2_/5% CO_2_ at 33–34 °C at a rate of 100 μL/min (Figure S2C). Slices were then incubated with 50 mM KCl or 500 μM METH buffer (base: K-H buffer), and all perfusates were collected every 5 min in chilled tubes containing 50 μL 0.1 M perchloric acid buffer. Samples were filtered through a 0.22 μm syringe filter, and the dopamine level was measured. Briefly, 20 μL the sample was injected into an HPLC EiCOMPAK SC-5ODS column (3 μm, 2.1 × 150 mm, Eicom, Tokyo, Japan) with 83% 0.1 M acetic acid-citric acid, 17% methanol, 190 mg/L sodium 1-octanesulfonate, and 5 mg/L EDTA·Na, pH 3.5 mobile phase for 10 min at a rate of 230 μL/min.

### 4.10. Mouse Primary Neuronal Culture

Neurons were prepared from the VTA and striatum of C57BL/6J mouse embryos (E16). After removing the cerebrum, the VTA and striatum were dissociated into single-cell suspensions with a cell strainer (100 μM pore size, BD Biosciences, San Jose, CA, USA). The cells were seeded on poly-L-lysine coated coverslips and plated at a density of 2.5 × 10^4^ cells per cm^2^ in neuron culture medium (Sumitomo Bakelite, Tokyo, Japan) with 5 μM Ara-C (Sigma-Aldrich, St. Louis, MO, USA) and 1× antibiotic-antimycotic (Invitrogen, Carlsbad, CA, USA). The cells were incubated in the culture medium in a humidified incubator at 37 °C and 5% CO_2_.

### 4.11. In Vitro CB1 Knockdown/Overexpression

We performed CB1 knockdown or overexpression in primary cultured neurons to study the relationship between CB1 and GAD67. CB1 human open reading frame (ORF) clone lentiviral particles (c-Myc tagged) were obtained from Origene (Rockville, MD, USA) for CB1 overexpression. Lentiviral particles (5 MOI) were added to primary cultured neurons at days in vitro (DIV) 5 and the neurons were incubated for 24 h for transduction. After transduction, the neurons were treated with control or JWH-210 (0.1 µM) for 24 h and analyzed by immunocytochemistry/immunofluorescence. Mouse control and CB1 shRNA lentiviral particles (2 MOI) were added to primary cultured neurons at DIV 5 and incubated for 16 h for transduction. Media was changed after transduction, and the neurons were incubated for another 48 h. After incubation, neurons were analyzed by immunocytochemistry/ immunofluorescence and qPCR.

### 4.12. Immunocytochemistry/Immunofluorescence

Immunocytochemistry/immunofluorescence was done as described previously [62]. The cultured cells were incubated with the following primary antibodies: β-tubulin III (TUJ1) (1:300, Abcam, Cambridge, MA, USA), CB1 (1:300, Abcam, Cambridge, MA, USA), GAD67 (1:300, Abcam, Cambridge, MA, USA), c-Myc (1:300, Abcam, Cambridge, MA, USA), and GFP (1:300, Abcam, Cambridge, MA, USA) at 4 °C overnight. Then, cells were incubated with secondary antibodies conjugated to Alexa Fluor 405, 488, or 594 (1:500, Invitrogen, Carlsbad, CA, USA) at RT for 1–2 h. Finally, cells were incubated with DAPI (2 mg/mL stock, 1:1000, Sigma-Aldrich, St. Louis, MO, USA) at RT for 5 min. Images were captured using a Leica DM5500B microscope and Leica DFC495 camera (Leica Microsystems, Wetzlar, Germany). LAS-AF (version 3.1.0 build 8587, Leica Microsystems, Wetzlar, Germany) software was used to merge single monochromatic fluorescent micrographs.

### 4.13. Quantitative Real-Time PCR (qPCR)

For mRNA quantification, total RNA was extracted from mouse brains using a total RNA extraction kit (iNtRON Biotechnology, Seongnam, Korea). Complementary DNA (cDNA) was synthesized from total isolated RNA using a SuperScript III first-strand synthesis kit (Invitrogen, Carlsbad, CA, USA). Quantitative real-time PCR was performed with an iCycler iQ5 Real-Time Detection System (Bio-Rad, Hercules, CA, USA) using SYBR GreenER qPCR SuperMix Universal (Invitrogen, Carlsbad, CA, USA) and the following primers: mouse CB1, forward 5′-GTACCATCACCACAGACCTCCTC-3′ and reverse 5′-GGATTCAGAATCATGAAGCACTCCA-3′; mouse GAD67, forward 5′-GTGCTGCTCCAGTGTTCTGCCATC-3′ and reverse 5′-AATCCCACAGTGCCCTTTGCTTTCC-3′; and mouse GAPDH, forward 5′-TGTCAAGCTCATTTCCTGGT-3′ and reverse 5′-CTTACTCCTTGGAGGCCATG-3′. Results were normalized to GAPDH and quantified relative to expression in control samples. For relative quantification, the 2^−^^ΔΔCT^ formula was used, where:

−ΔΔCT = (C_T,target_ − C_T,GAPDH_) experimental sample − (C_T,target_ − C_T,GAPDH_) control sample.

### 4.14. Western Blotting

Mice brain were prepared as previously described [63]. Briefly, mice brain homogenized with RIPA buffer (Thermo Fisher Scientific, Rockford, IL, USA), and incubated on ice for 60 min, and centrifuged at 13,000 rpm for 20 min at 4 °C. An equal amount of total protein (30 μg) was subjected to SDS-PAGE (12%), and the membranes were incubated with the following primary antibodies: CB1 (1:500, Abcam, Cambridge, MA, USA), GAD67 (1:1000, Abcam, Cambridge, MA, USA), β-actin (1:1000, Abcam, Cambridge, MA, USA), and GAPDH (1:500, Cell Signaling Technology, Danvers, MA, USA). The membranes were then incubated with horseradish peroxidase-conjugated anti-rabbit and anti-mouse secondary antibodies (1:5000, Sigma-Aldrich, St. Louis, MO, USA). Immunoreactivity was visualized with an ECL Plus detection system (GE Healthcare, Chicago, IL, USA). The relative density of the protein bands was analyzed with ImageJ.

### 4.15. Climbing Behavior

To study the role of dopamine receptor activity induced by GAD67 knockdown, climbing behavior was measured. Three days after GAD67 siRNA injection, mice were moved into cylindrical cages (diameter, 12 cm; height, 14 cm) with the floor and walls consisting of metal bars (0.2 cm diameter; separated by 1 cm gaps) and covered with a lid. After a 10-min acclimation period, apomorphine (1 mg/kg, i.p.) or vehicle (i.p.) was injected. The time spent for climbing was measured for 1, 10, 20, and 30 min after drug administration.

### 4.16. Immunohistochemistry/Immunofluorescence

Mice were euthanized by CO_2_ inhalation and perfused with phosphate-buffered saline (PBS, pH 7.4) with heparin and 4% para-formaldehyde (PFA) in PBS (pH 7.4) at the end of the behavior tests. The mice brains were processed and made into brain sections (10 μm) in the same way as in a previous study [63]. Before staining, the brain sections were air-dried for 3 h. After two 10-min washes in PBS (pH 7.4), the brain sections were incubated at 60 °C citrate buffer (10 mM citric acid, pH 7.4) for 30 min, incubated with the following primary antibodies: GAD67 (1:300, Sigma Aldrich, St. Louis, MO, USA), GFP (1:200, Abcam, Cambridge, MA, USA), dopamine receptor D1 (DRD1; 1:200, Novusbio, Centennial, CO, USA), and dopamine receptor D2 (DRD2; 1:200, Merck Millipore, Burlington, MA, USA) and secondary antibody conjugated to biotinylated goat anti-mouse IgG-horseradish peroxidase (HRP) (1:500, Santa Cruz, CA, USA) or Alexa Fluor 488 and 594 (1:500, Invitrogen, Carlsbad, CA, USA) [64]. The brain sections were evaluated on a light microscopy (Microscope Axio Imager.A2, Carl Zeiss, Oberkochen, Germany, ×200) or a confocal microscope (LSM980, Carl Zeiss, Oberkochen, Germany, ×200).

### 4.17. Ethical Approval of the Human Study

Ethical approval for the Stanley Brain Collection was obtained through the Institutional Review Board (IRB) of the Uniformed Services University of the Health Sciences, Bethesda, MD, who determined that IRB approval was not needed (during the collection period of 1998–2004) because the human subjects were deceased and all work was being done on de-identified specimens that were simply numbered. Consent to donate the specimens was obtained from next-of-kin and witnessed by two people who signed a form verifying the fact. Subsequently, the next-of-kin was contacted and interviewed to obtain further information about the deceased. These studies were carried out in accordance with the declaration of Helsinki, after approval by the Human Research Ethics Committee at the University of New South Wales (#HREC07261).

### 4.18. Human RNA-Seq Data

RNA-Seq data used in our previous study [65] was reanalyzed in this study. Briefly, the RNA-Seq data was generated from the human brain of 113 individuals with schizophrenia and unaffected controls. The samples are from the SMRI tissue collections; the Neuropathology Consortium (NPC), the Array Collection (AC), and the New Stanley Collection (NSC). Mapping the RNA-seq reads, quantifying the mapped reads and normalization of the mapped reads were performed as previously described [65].

### 4.19. Correlation Analysis between RNA-Seq Data and the Severity Ratings of Drug Use

Qualitative ratings (on a 0 to 5 scale) are used for severity of drug use of all SMRI cases [66]. Since the ratings of two cases were not available, the two samples were excluded in this analysis. Correlation analysis was performed between normalized RNA-seq data and the severity ratings of drug use. *p*-values less than 0.01 were considered significant.

### 4.20. Functional Annotation

DAVID (http://david.abcc.ncifcrf.gov/home.jsp, Accessed date: 9 October 2019) was used to identify the biological processes that were significantly over-represented by genes correlated with the severity of drug use in the frontal cortex. *p*-values less than 0.05 were considered significant.

### 4.21. Data Analysis

The data represent the mean ± standard error (S.E.). Data were analyzed with Pearson correlation test, Fisher’s exact test, Student’s *t*-test, one-way, two-way, three-way, and two-way repeated measures (RM) analysis of variance (ANOVA) followed by Bonferroni or Holm–Sidak *post-hoc t*-test using SigmaPlot 14 software (Systat Software, San Jose, CA, USA).

## 5. Conclusions

In the present study, we examined the effect of JWH-210, a CB1 agonist, on METH-induced reward. We found that pretreatment of JWH-210 augmented METH-induced drug reward in CPP test, and KCl-induced dopamine release or METH-induced dopamine efflux in mouse striatum. At this time, CB1 and GAD67 levels in mouse striatum were decreased by repeated administration of JWH-210. Interestingly, expression of GAD67 was affected by level of CB1. Based on these results, we measured METH-induced CPP score in mouse, which is CB1 or GAD67 knockdown in striatum. As a result, knockdown of CB1 or GAD67 increased METH-induced CPP. Moreover, in the human brain, mRNA level of *GAD67* was found to be decreased in drug users. Our study suggests that CB1 potentiates METH-induced CPP through inhibitory GABAergic regulation of dopaminergic neuronal activity.

## Data Availability

The data that support the findings of this study are available on request from the corresponding authors.

## Supplementary Materials

The following are available online at https://www.mdpi.com/article/10.3390/ijms221910486/s1.
