# Supplementary material for: Cannabinoid Receptor Type 1 Regulates Drug Reward Behavior via Glutamate Decarboxylase 67 Transcription
Source: Int J Mol Sci. 2021 Sep 28;22(19):10486. doi: 10.3390/ijms221910486 (PMC8508987; doi:10.3390/ijms221910486)
Supplement: Supplementary file 1 [file ijms-22-10486-s001.zip › ijms-1328388-supplementary.pdf]

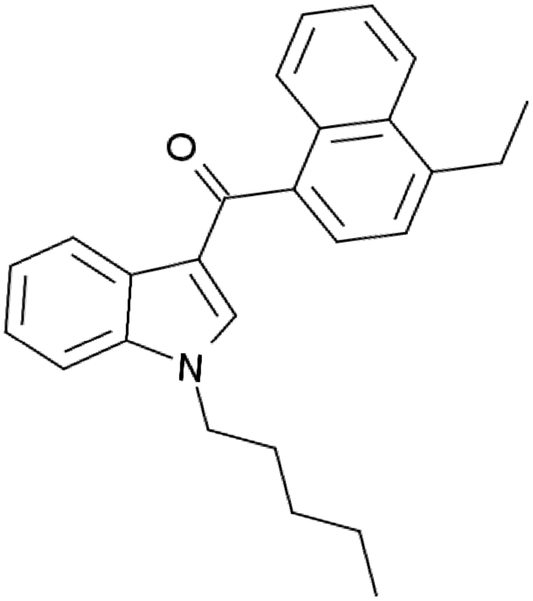

JWH-210

Figure S1. Chemical structure of JWH-210.

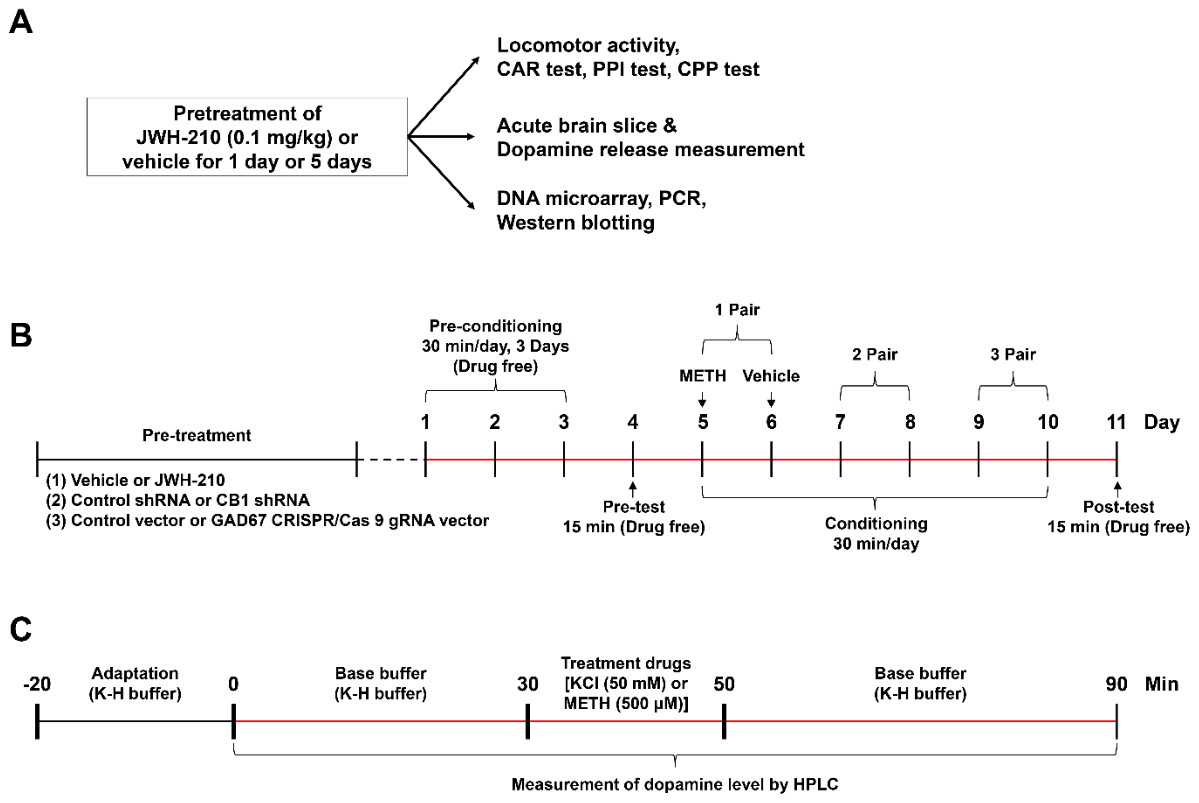

Figure S2. Timeline of behavioral studies. (A) Process of drug administration and behavior tests and molecular studies. (B) Diagrams illustrating the timeline of CPP procedures. (C) Diagrams illustrating the timeline of acute brain slice.

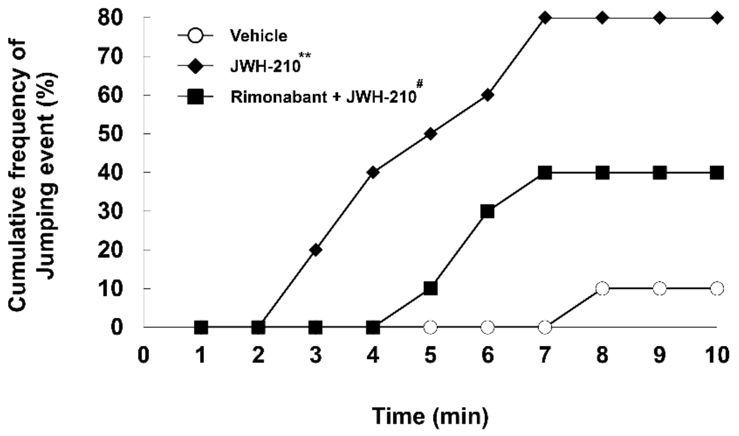

Figure S3. Effects of the CB1 antagonist on JWH-210-induced impulsivity. To clarify the CB1 mechanism of action on JWH-210-induced behavioral effects, we conducted the CAR test using rimonabant as the CB1 antagonist. Rimonabant hydrochloride was obtained from Sigma-Aldrich (St. Louis, MO, USA). The mice were divided into three groups: (1) Vehicle (vehicle + vehicle); (2) JWH-210 (vehicle + JWH-210); (3) Rimonabant (rimonabant + vehicle). The mice were treated with either vehicle (10% DMSO, 10% tween-80, 80% saline) or rimonabantm (1 mg/kg), followed by either vehicle or JWH-210 (0.1 mg/kg, i.p.), after 30 min once every day for 5 days. On day 6, CAR tests were performed as described in the Materials and Methods section. The cumulative frequency of jumping events (%) were expressed as the differences between vehicle group and other group curves, and were analyzed using one-way ANOVA [$F(2,27) = 10.983$, $p < 0.001$] followed by Bonferroni *post-hoc* t-test ($n = 10$). The jumping event was significantly increased in the JWH-210 group compared to that in the with vehicle-treated group, as shown in Figure 2D ($^{**}p < 0.01$), and in the rimonabant group ($^{\#}p = 0.021$); however, the difference between the rimonabant group and the vehicle group was not statistically significant ($p = 0.295$). One mouse in the vehicle group, eight out of 10 mice in the JWH-210 group, and four out of 10 mice in the rimonabant group jumped off.

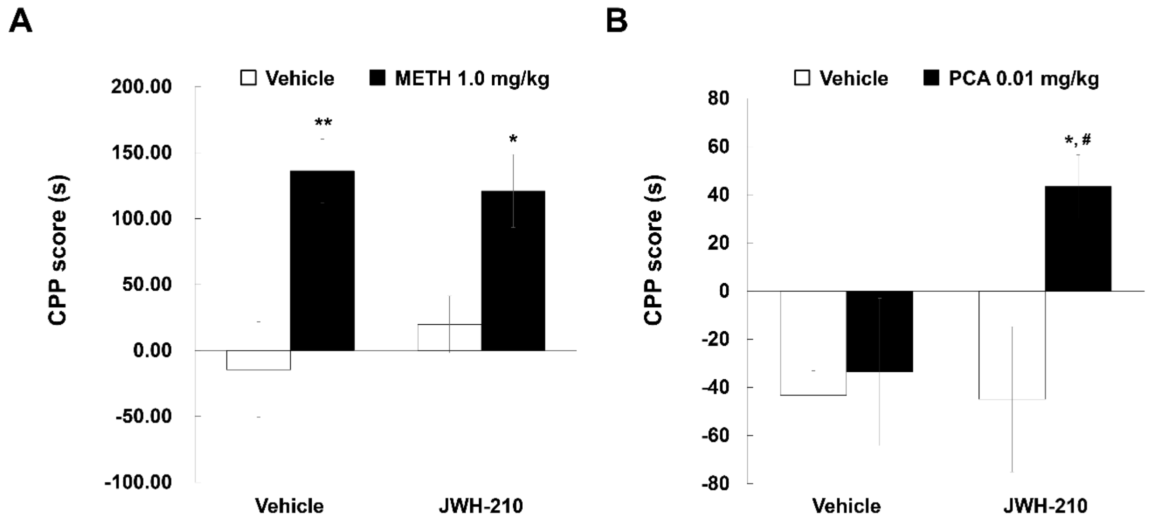

Figure S4. Effects of JWH-210 on METH (1.0 mg/kg)- or PCA (0.01 mg/kg)-induced CPP. To evaluate the effects of JWH-210 on the development of Pavlovian conditioning induced by METH (1 mg/kg) or PCA (0.01 mg/kg), the mice were treated with either a vehicle or JWH-210 (0.1 mg/kg, i.p.) once every day for 5 days, and then the CPP test was performed. (A) JWH-210 pretreatment itself did not affect the development of CPP, and METH-induced CPP was not significantly altered by pretreatment with JWH-210; the maximum effect was observed at 1 mg/kg METH. Data are expressed as the mean ± S.E. (n = 13) and were analyzed using two-way ANOVA [pretreatment drug condition: $F(1,41) = 0.110, p = 0.742$; CPP drug condition: $F(1,41) = 19.127, p < 0.01$; interaction: $F(1,41) = 0.740, p = 0.395$] followed by Bonferroni *post-hoc* t-test ($^{*}p < 0.05$ and $^{**}p < 0.01$ vs. each vehicle group). (B) PCA 0.01 mg/kg did not develop CPP, however, JWH-210 pretreatment significantly formed CPP on PCA 0.01 mg/kg. Data are expressed as the mean ± S.E. (n = 8) and were analyzed using two-way ANOVA [pretreatment drug condition: $F(1,28) = 2.677, p = 0.113$; CPP drug condition: $F(1,28) = 4.541, p = 0.042$; interaction: $F(1,28) = 2.907, p = 0.099$] followed by Bonferroni *post-hoc* t-test ($^{*}p < 0.05$ vs. each vehicle group; $^{\#}p < 0.05$ vs. vehicle/PCA group). METH: methamphetamine. PCA: para-chloroamphetamine.

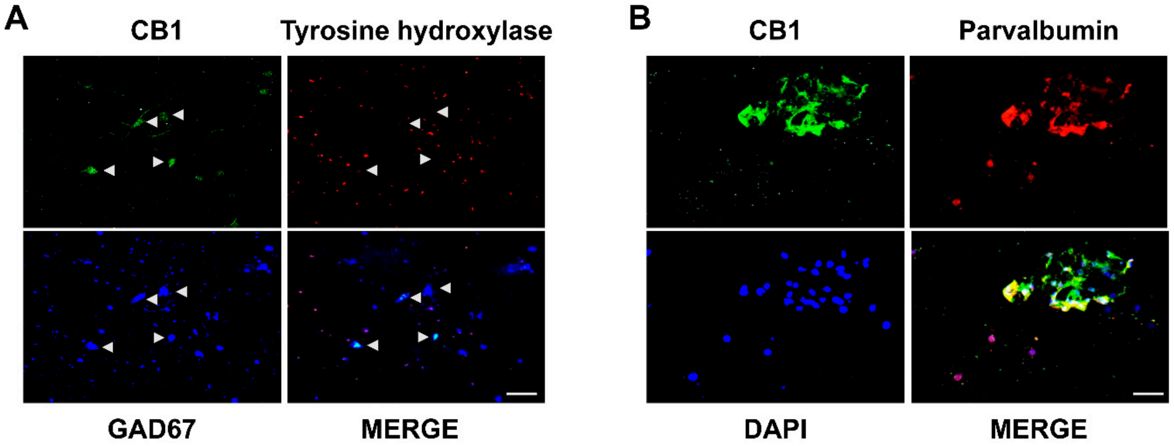

Figure S5. Immunocytochemistry results confirming the location of CB1 in GABAergic neurons. To confirm if CB1 is located in the GABAergic neurons or not in striatum, mouse primary cultured striatal neurons were cultured as Materials and Methods section. (A) In primary cultured striatal and VTA neurons, CB1 is mainly expressed in parvalbumin, known as GABAergic neurons marker, positive neurons. (B) In primary cultured striatal and VTA neurons, CB1 is not expressed in tyrosine hydroxylase, known as dopaminergic neurons marker, positive neurons. Closed arrowheads indicate both CB1 positive and tyrosine hydroxylase negative neurons. Original magnification is ×100. Scale bar: 100 μm.

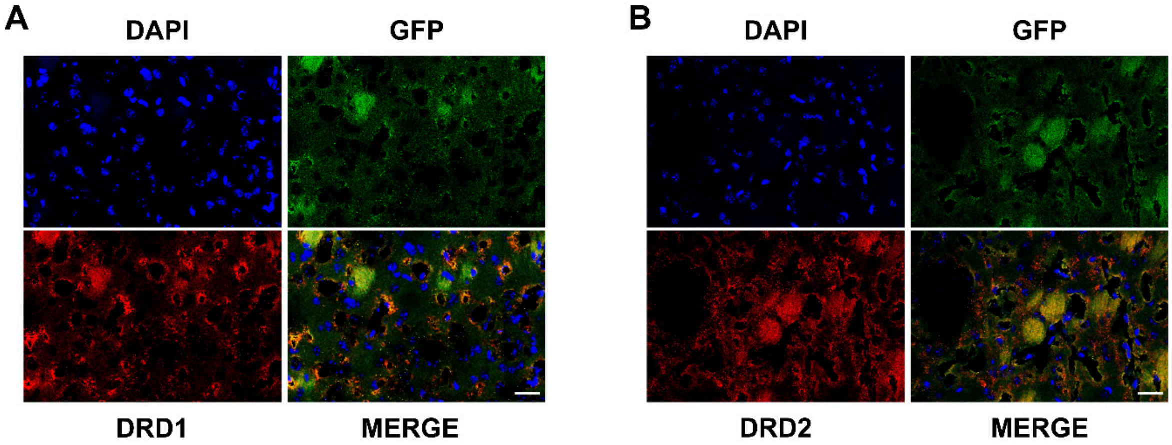

Figure S6. Immunohistochemistry results confirming the change of dopamine receptor type by GAD67 knockdown in mouse striatum. To confirm expression of dopamine receptor type by GAD67 knockdown, mouse striatum sections (10-μm-thick) were used for immunohistochemistry/immunofluorescence. (A) Representative sections are triple immunofluorescence staining with DRD1 (red), GFP (green), DAPI (blue). (B) Representative sections are triple immunofluorescence staining with DRD2 (red), GFP (green), DAPI (blue). Original magnification is ×200. Scale bar: 50 μm.

Table S1. Effects of JWH-210 on dopamine-related gene expression. To confirm the effects of JWH-210 administration on the regulation of gene expression related to dopamine, mice were treated with either a vehicle or JWH-210 (0.1 mg/kg, i.p.) once every day for 5 days. Next, the brain was excised from the skull. Total RNA was extracted from the mouse striatum using a total RNA extraction kit (iNtRON Biotechnology), and cDNA was synthesized from the RNA using a RT2 First Strand kit (Qiagen, Valencia, CA). The RT2 qPCR Master Mix was added to the cDNA, and the cDNA mixture was aliquoted into the wells of RT2 ProfilerTM PCR Array for the Mouse Dopamine & Serotonin Pathway (QIAGEN, Cat# PAMM-158Z). After PCR amplifications, the results were normalized to those of the control group and presented as fold change using the ΔΔCT method. The results were analyzed using Student's t-test [not significant (N.S.) vs. control group]. Administration of JWH-210 did not affect any major genes related to dopamine, such as *Th* (tyrosine hydroxylase), *Slc6a3* (dopamine transporter), *Slc18a2* (vesicular monoamine transporter 2), *Drd1a* (dopamine receptor D1a), *Drd2* (dopamine receptor D2), *Maoa* (monoamine oxidase A), and *Maob* (monoamine oxidase B).

Gene symbol	Description	Fold Change	P value
Th	Tyrosine hydroxylase	1.1131	N.S.
Slc6a3	Solute carrier family 6 (neurotransmitter transporter, dopamine), member 3; DAT	0.7817	N.S.
Slc18a2	Solute carrier family 18 (vesicular monoamine), member 2; VMAT2	0.9231	N.S.
Drd1a	Dopamine receptor D1A	0.6665	N.S.
Drd2	Dopamine receptor D2	1.2698	N.S.
Maoa	Monoamine oxidase A	0.7817	N.S.
Maob	Monoamine oxidase B	0.8495	N.S.

Table S2. Functional annotation. DAVID (http://david.abcc.ncifcrf.gov/home.jsp) was used to identify the biological processes that were significantly over-represented by genes correlated with the severity of drug use in the frontal cortex. P-values less than 0.05 were considered significant. The major biological processes (gene ontology) significantly enriched in the genes that correlated with the severity ratings.

Term	Count	Fold enrichment	P value
GO:0042493~response to drug	13	3.4	4.99E-04
GO:0010628~positive regulation of gene expression	12	3.6	5.13E-04
GO:0045893~positive regulation of transcription, DNA-templated	17	2.6	8.64E-04
GO:0035791~platelet-derived growth factor receptor-beta signaling pathway	3	47.3	0.002
GO:0000187~activation of MAPK activity	7	5.2	0.002
GO:0051607~defense response to virus	8	3.8	0.01
GO:0021915~neural tube development	4	10.5	0.01
GO:0001666~response to hypoxia	8	3.7	0.01
GO:0045944~positive regulation of transcription from RNA polymerase II promoter	23	1.8	0.01
GO:0007275~multicellular organism development	15	2.3	0.01
GO:0031076~embryonic camera-type eye development	3	21.5	0.01
GO:0001837~epithelial to mesenchymal transition	4	9.3	0.01
GO:0030177~positive regulation of Wnt signaling pathway	4	8.8	0.01
GO:0048566~embryonic digestive tract development	3	14.8	0.02
GO:0030193~regulation of blood coagulation	3	13.9	0.02
GO:0033189~response to vitamin A	3	13.1	0.02
GO:1902895~positive regulation of pri-miRNA transcription from RNA polymerase II promoter	3	11.8	0.03
GO:0001501~skeletal system development	6	3.5	0.03
GO:0042475~odontogenesis of dentin-containing tooth	4	5.7	0.03
GO:0070373~negative regulation of ERK1 and ERK2 cascade	4	5.4	0.04
GO:0060128~corticotropin hormone secreting cell differentiation	2	52.6	0.04
GO:0000188~inactivation of MAPK activity	3	9.5	0.04
GO:0001649~osteoblast differentiation	5	3.8	0.04
GO:0005981~regulation of glycogen catabolic process	2	39.4	0.05
GO:0001845~phagolysosome assembly	2	39.4	0.05
GO:0060129~thyroid-stimulating hormone-secreting cell differentiation	2	39.4	0.05
